# Application of machine learning models in predicting physical literacy in 4–6-year-old children: A comprehensive analysis of individual and family factors

**DOI:** 10.1371/journal.pone.0332997

**Published:** 2025-09-30

**Authors:** Xiaofen Wang, Ying Jiang

**Affiliations:** 1 Chengyi College, Jimei University, Xiamen, Fujian, China; 2 Fujian Institute of Education, Fuzhou, Fujian, China; Faculty of Physical Education and Sports at Pedagogical University of Maputo, MOZAMBIQUE

## Abstract

Physical literacy in children has become a significant research topic in both education and psychology. Recently, machine learning, as a cutting-edge AI technology, has started to play a crucial role in these fields. This study aimed to apply machine learning models to predict physical literacy in 4–6-year-old children and to comprehensively analyze the influence of individual and family factors. We evaluated the physical literacy of 1,734 children aged 4–6 and systematically examined the impact of both individual factors (such as gender, age, body type, sedentary behavior, screen time, moderate-to-vigorous physical activity (MVPA), sleep duration, and sleep quality) and family factors (such as parents’ education level, occupation, exercise frequency, support for children’s physical activity, household annual income, and family exercise environment) using various machine learning models. Results showed that the ensemble learning model achieved the best performance in predicting physical literacy, with an AUC of 86.2%. Among all predictive factors, mother’s exercise frequency, family exercise environment, and time spent on MVPA were identified as the most important. These findings provide new insights into enhancing children’s physical literacy and underscore the critical role of family environment and lifestyle in its development.

## Introduction

In the field of physical literacy research, British scholar Whitehead, drawing from monism, existentialism, and phenomenology, defined physical literacy as the combination of motivation, confidence, physical competence, knowledge, and understanding needed to maintain lifelong physical activity [1]. The Australian Physical Literacy Framework further breaks down physical literacy into physical, psychological, social, and cognitive domains [2]. Although various definitions exist, they all share a holistic view, emphasizing the psychological, physical, and cognitive attributes necessary for lifelong participation in physical activities [3]. Physical literacy, therefore, can be viewed as a continuous journey, where its components interact to foster lifelong engagement in physical activity, ultimately contributing to population health, well-being, and quality of life.

With the global spread of physical inactivity, particularly among younger children [4], fostering physical literacy has become a key area in promoting children’s health [5]. For children aged 4–6, who are at a critical stage in developing physical literacy, their rapid physical and cognitive growth offers an essential opportunity for early cultivation. Thus, focusing on this early life stage is crucial to ensuring their future active participation in physical activities and maintaining good health [6].

Recent studies have highlighted the importance of physical literacy. Integrating physical literacy into active school recesses has been shown to significantly improve children’s physical fitness and academic achievement [7]. A systematic review and meta-analysis also confirmed that physical literacy is significantly and positively associated with cardiorespiratory fitness in children and adolescents [8]. However, despite widespread recognition of the importance of physical literacy, research has primarily focused on adolescents and adults [9], leaving a noticeable gap in studies on preschool children aged 4–6. Additionally, the influence of family environment and parental behavior on children’s physical literacy has increasingly gained attention. Studies have shown that parents’ exercise habits, the family’s exercise atmosphere, and their support for children’s physical activity significantly impact children’s physical literacy, with objective measurements revealing a strong correlation between parents’ and children’s physical activity levels [10]. Moreover, the family exercise environment, especially aspects related to physical activity, such as parents’ exercise preferences and household setups, plays a critical role in shaping children’s physical behavior [11]. However, existing research has predominantly focused on school curricula [12,13] and teacher-related factors [14], with limited exploration of how the family environment directly influences early physical literacy development. Family factors, including parents’ attitudes, behaviors, and the home environment, have a direct and lasting influence, especially for children aged 4–6, who rely heavily on their families [15–17]. Therefore, expanding the scope of research to include family environment factors, particularly parental exercise habits, is essential to understanding their impact on preschool children’s physical literacy.

With the rapid advancement of data science and AI, machine learning has demonstrated considerable advantages in handling large-scale, complex data, and is gradually being applied in children’s health research [18]. Compared to traditional statistical methods, machine learning can identify more complex and hidden health risk factors, thereby improving data analysis accuracy and prediction reliability [19]. For example, Guerrero et al. used decision tree analysis to study Canadian children’s adherence to 24-hour movement guidelines during the COVID-19 pandemic, finding that parental perceptions significantly influenced children’s compliance with exercise guidelines [20]. Similarly, Bitew et al. employed various machine learning algorithms to predict malnutrition in under-five children in Ethiopia, with the xgbTree algorithm showing superior predictive ability in identifying key factors [21]. Xu and Sun used machine learning methods to explore the relationship between physical fitness and academic performance in primary school students, successfully predicting the correlation between these variables [22].

Despite the promising potential of machine learning in children’s health research, current studies remain in the early stages and are largely confined to specific applications. Particularly in preschool children’s physical literacy, the application of machine learning is still relatively limited, with related research mainly focusing on school-aged or older samples [23,24]. Moreover, the existing literature on physical literacy largely relies on traditional approaches [2].Most existing studies rely on single models (as above), missing out on the advantages of ensemble learning. By integrating results from multiple algorithms, ensemble learning enhances model stability and prediction accuracy [25], and recent evidence has demonstrated the advantages of ensemble learning approaches in children’s health [26]. However, the potential of ensemble learning models in handling complex, multidimensional data related to children’ s health has yet to be fully explored [27].

This study aimed to systematically analyze and compare the predictive effects of individual and family factors on physical literacy in preschool children aged 4–6 using ensemble machine learning models.

## Research methods

### Research subjects

This study employed a cross-sectional observational design with a stratified cluster sampling method to select participants from 18 kindergartens across nine cities in Fujian Province, including Fuzhou, Xiamen, Putian, Sanming, Quanzhou, Zhangzhou, Nanping, Longyan, and Ningde. In each city, two kindergartens were selected to represent both main urban districts and urban–rural fringe areas based on administrative divisions [28], with “main urban districts” referring to the core urban areas and “urban–rural fringe areas” referring to the transitional zones between urban and rural areas. One class each from the large, medium, and small age groups was randomly selected from each kindergarten. A total of 1,885 children were initially recruited. After excluding 151 questionnaires due to missing data or patterned responses, 1,734 valid cases were retained for analysis. The required sample size was evaluated with reference to two principles: (1) scale development best practices suggest a minimum of 5–10 participants per item [29], and given that this study employed the 30-item PL-C Quest and the 23-item Family Environment Scale, the minimum required sample size would be 265–530; (2) the events per variable (EPV) principle recommends at least 10 outcome events per predictor variable [30], and with 16 predictors, the minimum required sample size would be about 160. The actual sample size of 1,734 preschool children far exceeded these requirements, ensuring sufficient statistical power and robustness. Parents were invited to participate in the study, and informed consent was obtained before they completed the survey. The inclusion criteria were children aged 4–6 years, excluding those with physical developmental disabilities, intellectual disabilities, genetic disorders, or severe organic diseases. The data collection was conducted between November 2023 and May 2024.This study was ethically reviewed and approved by the Scientific Research Promotion Department at Chengyi College, Jimei University.

### Research tools

#### Basic information questionnaire.

We designed a “Basic Information Questionnaire” tailored to the study’s objectives. This questionnaire gathered data on children’s personal information, including gender, age, kindergarten location, body type, sedentary behavior, screen time, moderate-to-vigorous physical activity (MVPA) time, sleep duration, and sleep quality. It also collected family-related information, such as parents’ education levels, occupations, exercise frequency, support for children’s physical activity, and household annual income. Parents completed the questionnaire on behalf of their children. Sedentary behavior and MVPA were assessed using the International Physical Activity Questionnaire—Short Form (IPAQ-SF) [31], a widely recognized and validated instrument. Previous studies have demonstrated its reliability and validity in the Chinese population [32]. The questionnaire consists of seven items, covering physical activity and sedentary time. It measures the weekly frequency (days per week) and daily duration (minutes per day) of low-intensity, moderate-intensity, and vigorous-intensity physical activities, as well as daily sedentary time (minutes per day) over the past seven days. The average daily MVPA time was calculated as “(MPA frequency × duration + VPA frequency × duration)/7.” Furthermore, drawing on both international evidence [33,34] and domestic preschool research [35], this study categorized children into sedentary (>6 hours/day) and non-sedentary (≤6 hours/day) groups based on their daily sedentary time.

For parental exercise frequency, parents reported the number of times per week they engaged in physical activity, categorized as“Never,” “1–2 times/week,” and “≥3 times/week.”The level of parental support for children’s physical activity was assessed separately for fathers and mothers, with reference to previous research [36]. In this study, parental support was categorized into three levels: High Support (e.g., actively participating in the child’s physical activity), Medium Support (e.g., providing encouragement and logistical support), and Low Support (e.g., seldom engaging in or promoting physical activity participation).

#### Physical literacy in children questionnaire (PL-C Quest).

The study employed the PL-C Quest, developed by Sport Australia, to assess the physical literacy of children aged 4–6 years [37,38]. This is the world’s first pictorial self-assessment tool designed for young children to evaluate their physical literacy. The questionnaire covers four domains—physical, psychological, social, and cognitive—with 30 items in total. Children were assessed through face-to-face interviews using images, and their responses were scored on a four-point scale. Higher total scores indicated better self-perceived physical literacy. The PL-C Quest has been validated for reliability and feasibility in children aged 4–12 in China [39], showing strong test-retest reliability (total scale: *r* = 0.90) and good to excellent internal consistency (total scale: *α* = 0.94). In this study, the Cronbach’s alpha for the PL-C Quest was 0.949, with domain-specific alphas ranging from 0.859 to 0.896. In physical literacy research, percentile-based methods are commonly used to distinguish groups at different relative levels [40,41]. In line with the “Healthy China 2030” Planning Outline, which set a national goal of achieving a 20% health literacy rate among residents by 2020 [42], this study classified children based on their PL-C Quest scores, with the top 20% classified as the “high physical literacy” group and the remaining 80% as the “needs attention” group. Although health literacy and physical literacy are not entirely identical constructs, international curriculum policies frequently locate them within the same Health and Physical Education (HPE) framework and pursue them under shared holistic health goals [43]. It should be noted that being in the “needs attention” group does not indicate poor physical literacy but rather reflects that there is room for improvement.

#### Family environment scale on motor development for pre-school urban children.

We used the Family Environment Scale on Motor Development for Pre-school Urban Children, developed by Hua Jing et al. [44], to assess the family exercise environment. This parent-reported scale evaluates four dimensions: outdoor space, indoor space, toys (hardware environment), and parenting style (software environment), with 23 items in total. Higher scores reflect a better family exercise environment [45]. The scale has demonstrated good reliability (Cronbach’s *α* = 0.875) and validity, with acceptable fit indices (*χ*^*2*^/*df* = 4.810, GFI = 0.949, RMSEA = 0.046) [44]. In our study, the Cronbach’s alpha for this scale was 0.933.

### Quality control

Before the survey, researchers involved in the assessment of children’s physical literacy received standardized training. Prior to data collection, all parents signed an informed consent form before completing the questionnaire and allowing their children to be assessed. The form clearly outlined the purpose of the study, the evaluation process, and confidentiality measures, ensuring that parents participated voluntarily with full knowledge of the study details. It also ensured that parents understood the requirements and guidelines for completing the questionnaire. The questionnaires were reviewed by researchers, with strict exclusion of those that did not meet logical requirements, contained missing answers, or exhibited patterned responses. The data were entered into an Epidata database using a double-entry by two individuals to ensure data accuracy and correctness.

### Statistical methods

Data analysis was conducted using SPSS 21.0 and Python 3.6. Categorical data were summarized as frequencies and percentages, with group comparisons performed using chi-square tests. Normally distributed continuous data were presented as mean ± standard deviation and compared using *t*-tests. Non-normally distributed data were expressed as median (P25, P75) and compared using the Mann-Whitney *U* test. Statistical significance was set at *P* < 0.05. Sixteen significant factors from univariate analysis were selected for further modeling using eXtreme Gradient Boosting (XGBoost), Logistic Regression (LR), Random Forest (RF), and Support Vector Machine (SVM) algorithms. To account for potential clustering effects due to the stratified cluster sampling design, five-fold cross-validation was employed during model training and validation [46].Hyperparameter tuning was also conducted to optimize performance.The validation set comprised 20% of the data. XGBoost was implemented using Python’s xgboost 2.1.0, while LR, RF, and SVM were implemented using sklearn 1.5.0. Model performance was evaluated using receiver operating characteristic (ROC) curves, area under the curve (AUC), sensitivity, specificity, accuracy, and F1 score.

Single models can sometimes overfit the training data, especially with small or noisy datasets. Ensemble learning, which combines multiple models, can mitigate this risk by improving overall accuracy and reducing overfitting. This study employed a stacking ensemble method, combining the outputs of the three top-performing models based on AUC to create a final ensemble model, which was then tested on the prediction set.

ROC curves were used to assess the discriminatory ability of the models, with AUC values closer to 1 indicating better performance. The F1 score, which balances precision and recall, was used to evaluate model sensitivity, with a score of 1 indicating optimal performance and 0 indicating poor performance.

Feature importance for each model was calculated using SHAP (SHapley Additive exPlanations) values [47]. This method is based on Shapley value theory from cooperative game theory and quantifies the contribution of each feature to the model prediction by computing the weighted average of its marginal contributions across all possible feature subsets.

## Result

### General information

This study included 1,734 children aged 4–6 years from 18 kindergartens across nine cities in Fujian Province. Among the participants, 950 were boys (54.8%) and 784 were girls (45.2%). The majority were 6 years old (42.3%). Regarding kindergarten location, 1,031 children (59.5%) were from main urban districts and 703 (40.5%) were from urban–rural fringe areas. Body type was classified based on the Chinese national standard Growth Standards for Children Under 7 Years (WS/T 423–2022) [48], in which thinness and severe thinness were grouped as “Thin,” overweight, obesity, and severe obesity were grouped as “Overweight,” and the remainder were classified as “Normal.” Based on this classification, most children (74.0%) fell into the Normal BMI range. Notably, 80.3% of the children were reported to engage in low levels of sedentary behavior, and 65.3% were reported to have good sleep quality. Regarding the parents, 36.9% of fathers had a bachelor’s degree or higher, and most were self-employed (31.8%). The majority of fathers exercised 1–2 times per week (35.8%) and were generally supportive of their children’s physical activities (73.4%). For mothers, 36.7% had a bachelor’s degree or higher, and most worked in public institutions or mid-level management roles (29.9%). Interestingly, 38.1% of mothers did not exercise, although 75.3% were supportive of their children’s physical activities. The highest reported household income was 200,000 RMB or more, accounting for 27.0% of the families.

### Descriptive statistics of children’s physical literacy scores

The average physical literacy score for children aged 4–6 was 84.80 ± 14.26, with an average score per item of 2.83 ± 0.48. The mean scores across the domains were as follows: physical (2.62 ± 0.55), psychological (2.85 ± 0.53), social (3.17 ± 0.54), and cognitive (2.97 ± 0.55). The domain scores ranked from highest to lowest were: social, cognitive, psychological, and physical. Detailed statistics are provided in Table 1.

**Table 1:** Descriptive statistics of children’s physical literacy scores (n = 1,734).

Domain Name	Minimum	Maximum	Mean	Standard Deviation
Physical Domain	1	4	2.62	0.55
Psychological Domain	1	4	2.85	0.53
Social Domain	1	4	3.17	0.54
Cognitive Domain	1	4	2.97	0.55
Overall Physical Literacy	1	4	2.83	0.48

### Feature extraction

One-way analysis revealed significant differences between the “high physical literacy” group and the “needs attention” group across various factors, including age, body type, sedentary behavior, screen time, MVPA time, sleep duration, sleep quality, father’s occupation, father’s exercise frequency, father’s support for children’s physical activity, mother’s education level, mother’s occupation, mother’s exercise frequency, mother’s support for children’s physical activity, household annual income, and family exercise environment (**P* *< 0.05). The detailed comparisons are provided in Table 2.

**Table 2:** Comparison of features between high physical literacy and needs attention groups [*x*± *s*,n(%)].

Survey Content	Group	Number of People	Needs Attention Group (n = 1394)	High Physical Literacy Group (n = 340)	*t*/*χ²* Value	*P*-Value
Gender	Male	950	778 (81.9)	172(18.1)	*χ²* = 3.009	0.082
	Female	784	616(78.6)	168(21.4)		
Age (years)	4~	423	364(86.1)	59(13.9)	*χ²* = 12.526	0.002**
	5~	577	461(79.9)	116(20.1)		
	6~	734	569(77.5)	165(22.5)		
Kindergarten Location	main urban districts	1031	820(79.5)	211(20.5)	χ² = 1.187	0.276
	urban–rural fringe areas	703	574(81.7)	129(18.3)		
Body Type(Based on BMI Categories)	Thin	433	373(86.1)	60(13.9)	*χ²* = 13.364	0.001**
	Normal	1283	1005(78.3)	278(21.7)		
	Overweight	18	16(88.9)	2(11.1)		
Sedentary Behavior	Sedentary (>6 h/day)	342	305(89.2)	37(10.8)	*χ²* = 20.878	0.000***
	Non-Sedentary (≤6 h/day)	1392	1089(78.2)	303(21.8)		
Screen Time (Minutes)		1734	65.24 ± 58.53	57.45 ± 79.77	*t* = 2.036	0.042*
MVPA Time (Minutes)		1734	29.09 ± 35.15	50.87 ± 45.66	*t* = −8.220	0.000***
Sleep Duration (Hours)		1734	10.57 ± 1.37	10.78 ± 1.24	*t* = −2.664	0.008**
Sleep Quality	Good	1133	864(76.3)	269(23.7)	*χ²* = 35.460	0.000***
	Average	565	498(88.1)	67(11.9)		
	Poor	36	32(88.9)	4(11.1)		
Father’s Educational Level	Junior High or Below	319	266(83.4)	53(16.6)	*χ²* = 4.659	0.199
	High School/Technical School	389	318(81.7)	71(18.3)		
	College	386	311(80.6)	75(19.4)		
	Bachelor’s Degree or Above	640	499(78.0)	141(22.0)		
Father’s Occupation	Unemployed	45	37(82.2)	8(17.8)	*χ²* = 26.898	0.000***
	Self-employed	552	457(82.8)	95(17.2)		
	General Staff	412	354(85.9)	58(14.1)		
	Public Sector/Intermediate Management/Technical Personnel	464	353(76.1)	111(23.9)		
	Government Official/Senior Management/Technical Expert	147	102(69.4)	45(30.6)		
	Other	114	91(79.8)	23(20.2)		
Father’s Exercise Frequency (Times/Week)	Never	608	538(88.5)	70(11.5)	*χ²* = 112.038	0.000***
	1~	620	528(85.2)	92(14.8)		
	≥3	506	328(64.8)	178(35.2)		
Father’s Support for Children’s Physical Activity	High	1272	958(75.3)	314(24.7)	*χ²* = 78.095	0.000***
	Medium	386	364(94.3)	22(5.7)		
	Low	76	72(94.7)	4(5.3)		
Mother’s Educational Level	Junior High or Below	295	254(86.1)	41(13.9)	*χ²* = 20.620	0.000***
	High School/Technical School	364	304(83.5)	60(16.5)		
	College	439	359(81.8)	80(18.2)		
	Bachelor’s Degree or Above	636	477(75.0)	159(25.0)		
Mother’s Occupation	Unemployed	304	247(81.3)	57(18.7)	*χ²* = 11.926	0.036*
	Self-employed	288	241(83.7)	47(16.3)		
	General Staff	494	411(83.2)	83(16.8)		
	Public Sector/Intermediate Management/Technical Personnel	518	393(75.9)	125(24.1)		
	Government Official/Senior Management/Technical Expert	41	31(75.6)	10(24.4)		
	Other	89	71(79.8)	18(20.2)		
Mother’s Exercise Frequency (Times/Week)	Never	661	590(89.3)	71(10.7)	*χ²* = 127.554	0.000***
	1~	653	544(83.3)	109(16.7)		
	≥3	420	260(61.9)	160(38.1)		
Mother’s Support for Children’s Physical Activity	High	1306	979(75.0)	327(25.0)	*χ²* = 99.871	0.000***
	Medium	378	369(97.6)	9(2.4)		
	Low	50	46(92.0)	4(8.0)		
Household Annual Income (10,000 RMB)	<7	306	264(86.3)	42(13.7)	*χ²* = 33.056	0.000***
	7~	359	308(85.8)	51(14.2)		
	12~	306	251(82.0)	55(18.0)		
	16~	294	231(78.6)	63(21.4)		
	≥20	469	340(72.5)	129(27.5)		
Family Exercise Environment		1734	3.39 ± 0.65	4.19 ± 0.65	*t* = −20.628	0.000***

The *χ*^*2*^ value refers to the Chi-square test statistic, the *t* value refers to the t-test statistic, and the *P*-value indicates the significance level (*P* < 0.05 indicates statistical significance). Percentages are calculated within each row category using the “Number of People” as the denominator. *p < 0.05, **p < 0.01, ***p < 0.001.

### Model prediction results

**Fig 1:**
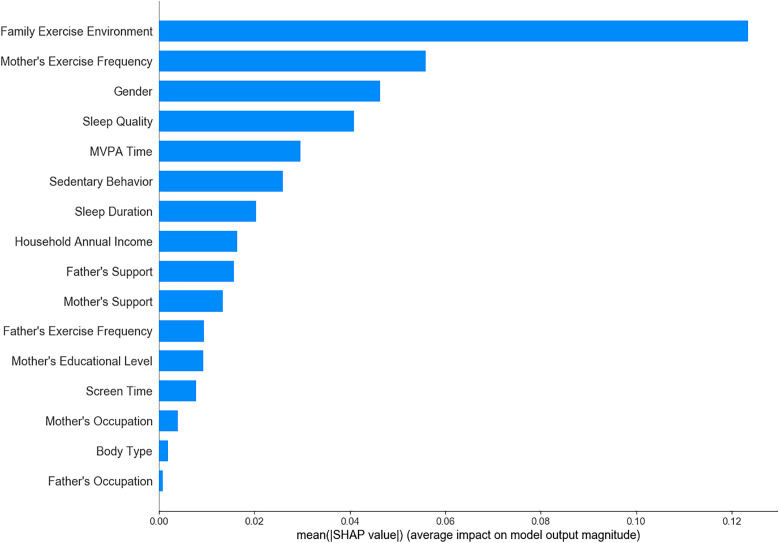
Feature importance ranking in LR model.

**Fig 2:**
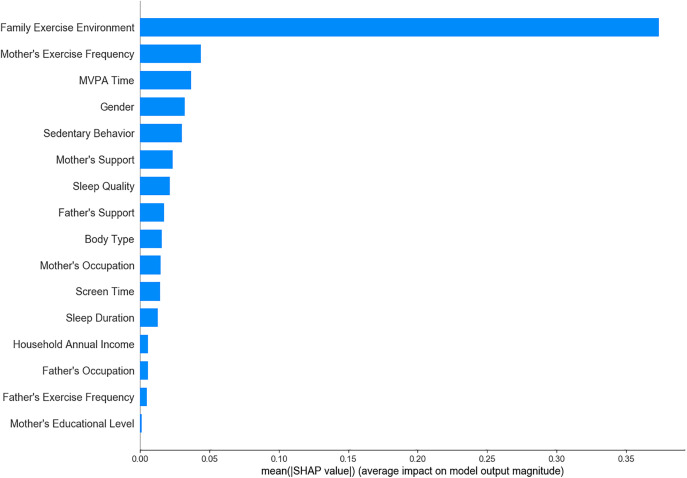
Feature importance ranking in SVM model.

**Fig 3:**
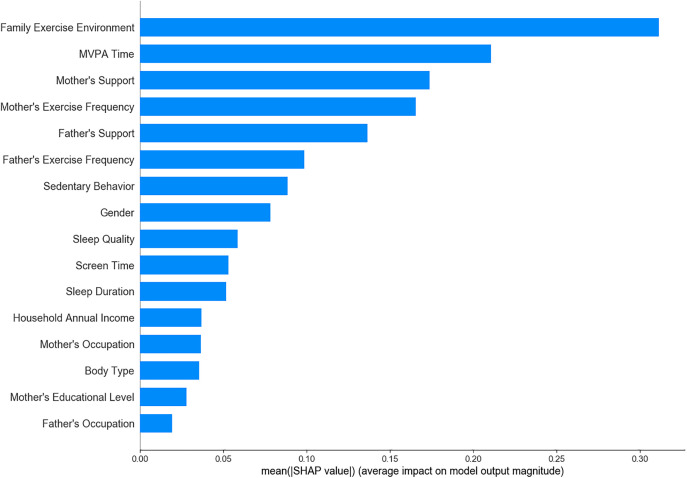
Feature importance ranking in XGBoost model.

**Fig 4:**
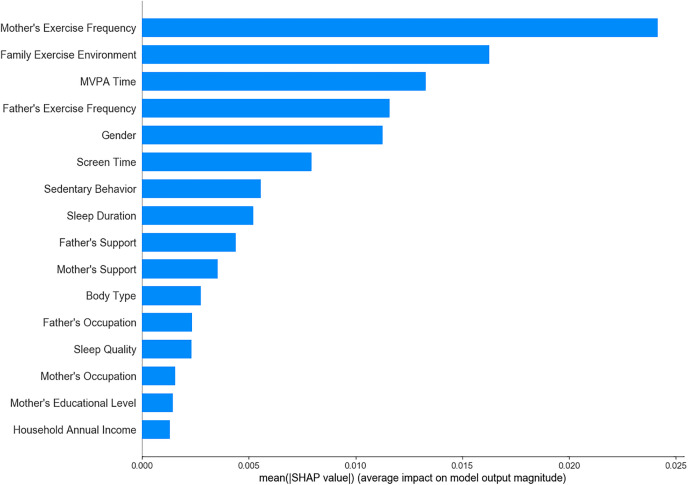
Feature importance ranking in RF model.

**Fig 5:**
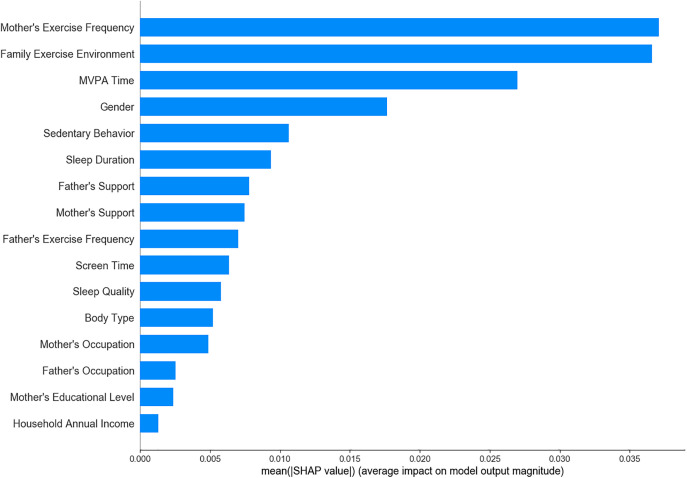
Feature importance ranking in ensemble learning model.

Among the 19 variables initially considered, 16 significant variables were identified through univariate analysis and were subsequently analyzed using LR, SVM, XGBoost, RF, and ensemble learning models to determine feature importance. The results showed that maternal exercise frequency, family exercise environment, and children’s MVPA time consistently emerged as key predictive factors across all models (Figs 1–5). The ensemble learning model, which integrated the strengths of multiple models, significantly improved prediction accuracy and robustness, reaffirming the critical impact of these factors on children’s physical literacy. Additionally, the ensemble learning model highlighted the importance of age, sedentary behavior, sleep duration, and parental support for children’s exercise. In contrast, factors such as parents’ occupation, household annual income, and children’s body type had relatively lower predictive power across the models (Fig 5).

### Model performance and comparison

In this study, the 16 significant variables were analyzed using four machine learning models, with 5-fold cross-validation for training and validation, and 20% of the data used as the test set. The models were trained with specific parameters: XGBoost was set with a binary classification objective, a learning rate of 0.05, max tree depth of 3, min child weight of 2, L2 regularization of 3, a feature sampling ratio of 0.2, and 100 estimators. The RF model used the Gini criterion, max tree depth of 4, and 100 estimators. SVM had a regularization factor (C) of 1, an rbf kernel, and a tolerance of 0.01. LR had a regularization factor (C) of 1, with max iterations of 500, and used the lbfgs solver.

The performance of each model is shown in Tables 3 and 4. The ensemble learning model consistently outperformed the individual models, particularly in AUC, specificity, accuracy, and F1 score. For the training set, the RF model had the highest AUC of 0.879 and an F1 score of 0.82, followed closely by XGBoost with an AUC of 0.870 and an F1 score of 0.80. The SVM and LR models showed weaker performance with AUCs of 0.847 and 0.831, and F1 scores of 0.79 and 0.76, respectively. In the test set, the ensemble learning model achieved the best results, with an AUC of 0.862, specificity of 0.829, accuracy of 0.816, and an F1 score of 0.83, demonstrating its superior ability to handle complex data and enhance model robustness. XGBoost and RF models performed similarly in the test set, both achieving AUCs of 0.858 and F1 scores of 0.80 and 0.81, respectively. The SVM and LR models lagged behind, with AUCs of 0.848 and 0.834, and F1 scores of 0.79 and 0.78, respectively. Overall, the ensemble learning model showed the best performance across all metrics, particularly in the test set, indicating its higher applicability and stability in predicting children’s physical literacy. While XGBoost and RF models also demonstrated relatively good predictive performance, they were still outperformed by the ensemble model. The SVM and LR models faced certain limitations in processing this type of data, resulting in slightly lower predictive performance.

**Table 3:** Training set model performance evaluation (N = 1,734).

Model	AUC	Sensitivity	Specificity	Accuracy	F1 Score
LR	0.831	0.788	0.729	0.740	0.76
SVM	0.847	0.791	0.761	0.767	0.79
XGBoost	0.870	0.806	0.777	0.783	0.80
RF	0.879	0.788	0.809	0.805	0.82
Ensemble Learning	0.878	0.766	0.813	0.804	0.82

**Table 4:** Test set model performance evaluation (N = 1,734).

Model	AUC	Sensitivity	Specificity	Accuracy	F1 Score
LR	0.834	0.791	0.746	0.755	0.78
SVM	0.848	0.821	0.754	0.767	0.79
XGBoost	0.858	0.791	0.786	0.787	0.80
RF	0.858	0.761	0.796	0.791	0.81
Ensemble Learning	0.862	0.761	0.829	0.816	0.83

**Fig 6:**
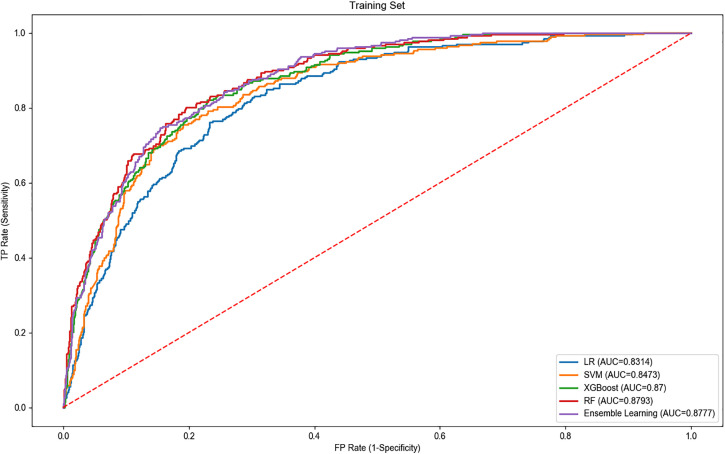
ROC curves of different models on training set.

**Fig 7:**
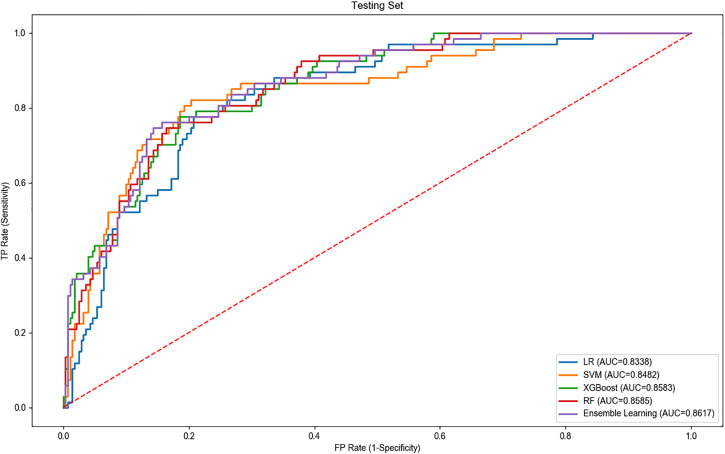
ROC curves of different models on test set.

The ROC curves for each model are illustrated in Figs 6 and 7.

## Discussion

This study assessed the physical literacy of 1,734 children aged 4–6 years in Fujian Province and revealed three key findings. First, children’s overall physical literacy level was relatively low, with notable variations across domains; the social domain achieved the highest scores, whereas the physical domain scored the lowest. Second, ensemble learning models outperformed single algorithms, underscoring their advantages in prediction accuracy and robustness. Third, mother’s exercise frequency, the family exercise environment, and children’s MVPA time consistently emerged as the most influential predictors of children’s physical literacy.

### Overall low levels of children’s physical literacy, with variations across domains

The findings of this study indicate that the overall physical literacy score for children in Fujian Province was 84.80, with an average item score of 2.83, slightly above the midpoint of 2.5. When compared with recent validation studies of the PL-C Quest in Chinese children, this score is relatively low. For example, one study reported a mean total score of 96.76 among 642 children aged 6–12 years [49], while another study reported a mean overall score of 98.8 in a larger sample of 1,870 children aged 4–12 years [39]. The substantially lower score observed in our sample suggests that children’s physical literacy levels in Fujian Province are generally low, highlighting a considerable gap compared to national reference data and indicating significant room for improvement. Among the four assessed domains, the social domain had the highest score, while the physical domain had the lowest. The high score in the social domain reflects that children display good ethical behavior, cooperation skills, and respect for different cultures and values during physical activities, indicating a solid understanding of socially expected physical literacy. Research shows that social relationships significantly influence behavior and beliefs, especially in children, where positive sports experiences heavily rely on social support [50]. Encouragement, respect, and understanding in physical activities can enhance children’s participation and confidence, thereby fostering continuous involvement in sports. Such social support not only enhances physical literacy but also contributes positively to overall development. However, this study revealed that children aged 4–6 scored the lowest in the physical domain, with an average score of 2.62, indicating deficiencies in motor skills and physical fitness. The physical domain is fundamental to children’s daily life and sports participation [50], and its importance for their future physical development cannot be overlooked. Previous studies have highlighted that children’s free time is often spent sitting, either studying or engaging in screen-based activities, with insufficient time dedicated to physical activities [51]. This trend may lead to a decline in overall physical literacy, particularly in physical ability, daily behavior, and psychological motivation and confidence [52]. Therefore, this study recommends a focus on improving the physical domain by encouraging children to increase their physical activity, thereby enhancing their fitness and motor skills and ultimately improving their overall physical literacy.

### Superior predictive performance of ensemble learning models for children’s physical literacy

By combining multiple models, including LR, SVM, XGBoost, and RF, this study represents the first application of ensemble learning methods in the field of children’s physical literacy, with the goal of improving prediction accuracy and model robustness. This study utilized five machine learning models—LR, SVM, XGBoost, RF, and Ensemble Learning—to train and validate 16 significant factors for constructing an efficient predictive model for physical literacy in children aged 4–6. The results showed that the Ensemble Learning model outperformed the others across key metrics, including AUC, specificity, accuracy, and F1 score, indicating its clear advantage in prediction accuracy and robustness. Compared to existing research, this study further validates the application of machine learning in predicting children’s physical literacy. Previous studies often relied on single algorithms. For instance, the XGBoost ensemble algorithm has been employed to develop an early health prediction framework [53], and it has also been used for assisting in orthopedic disease classification and prediction [54]. The RF algorithm has been utilized to predict adverse health events, demonstrating its potential in medical risk prediction [55]. In this study, the Ensemble Learning model not only exceeded single algorithms in predictive accuracy but also showed greater robustness. This finding is consistent with results demonstrating the effectiveness and adaptability of Ensemble Learning in processing complex data in dynamic multi-objective optimization [56]. Moreover, a review further supported the superiority of Ensemble Learning in disease prediction across various datasets [57].

### Key predictors of children’s physical literacy

This study evaluated the physical literacy of 1,734 children aged 4–6 and analyzed multiple individual and family factors using various machine learning models. The results showed that while the importance of different features varied across models, mother’s exercise frequency, the family exercise environment, and children’s MVPA time were consistently identified as the most critical predictors, underscoring their significant roles in the development of children’s physical literacy.

The Ensemble Learning model demonstrated the highest predictive capability with an AUC of 86.2%. This result indicates that leveraging multiple machine learning models enables a more comprehensive assessment and prediction of the factors influencing children’s physical literacy. Specifically, mother’s exercise frequency emerged as the most important predictor, with higher mother’s exercise frequency correlating with a higher likelihood of children being in the high physical literacy group (*P* < 0.05). This finding aligns with previous research [58], which reported that preschool children’s physical activity is closely linked to their mothers’ activity levels, with each additional minute of maternal exercise boosting preschool children’s MVPA participation by 10%. Although existing research has not deeply explored the relationship between maternal exercise and children’s physical literacy development, this study highlights the unique influence of maternal exercise frequency. According to the family influence model [59,60], a mother’s exercise behavior can directly impact her child’s physical activity and literacy through role modeling, emphasizing the crucial role of mothers in nurturing physical literacy. Given that previous studies have shown that mothers generally have low activity levels [61], future interventions should focus on increasing maternal physical activity to enhance children’s physical literacy.

Additionally, the family exercise environment was validated across multiple models as a key factor, with children in better exercise environments at home being more likely to achieve superior physical literacy (*P* < 0.05). A favorable family exercise environment not only provides sufficient space and resources for physical activities but also lays a solid foundation for the development of physical literacy. This finding aligns with existing research emphasizing the influence of family environments on children’s health behaviors, reinforcing the importance of creating a supportive exercise atmosphere for children. According to ecological systems theory, the family is the primary environment for child development, influencing physical literacy through individual, parental, and environmental factors. Although many studies focus on the roles of kindergartens and communities [62], research on the relationship between the family exercise environment and children’s physical literacy remains limited. Qualitative studies have pointed out that a lack of family exercise space hinders children’s participation in physical activities, especially self-initiated exercises, while encouraging more screen time [63]. Another review highlighted the critical role of the home environment in shaping children’s activity levels [64]. Our findings are consistent with these results. Additionally, a cross-sectional study on Chinese preschool children found that the family environment significantly impacts the scientific fitness literacy of both preschool and school-aged children [65]. Thus, the family exercise environment is an essential factor in cultivating children’s physical literacy.

Moreover, children’s MVPA time was consistently identified as an important predictor across all models, with children in the“high physical literacy” group having significantly more MVPA time than those in the “needs attention” group (*P* < 0.001). This finding suggests that children’s physical literacy is directly influenced by their level of physical activity. The study also identified sedentary behavior as a negative predictor, which aligns with related research. Studies have shown that physical literacy is associated with both physical activity and sedentary behavior [66]. For example, a study on Canadian children found that those who met the daily guideline of 60 minutes of moderate to vigorous physical activity scored higher in physical literacy, particularly in physical competence, motivation, confidence, and knowledge/skills [67]. This relationship may be due to the impact of exercise on cardiovascular health. Research by Lang et al. found a strong correlation between children’s cardiovascular health and their physical literacy and its components [5]. Lima R A et al. also highlighted a positive relationship between physical activity and motor competence, with cardiovascular endurance potentially acting as a mediator [68]. As previous studies have emphasized, physical literacy is not only a prerequisite for physical activity but also developed through it [69]. Therefore, we believe that insufficient physical activity and prolonged sedentary behavior are likely associated with lower physical literacy levels. Reducing sedentary time and replacing it with physical activity may be an effective strategy for improving children’s physical literacy [67]. Additionally, earlier studies have shown that prolonged sedentary behavior is negatively associated with cardiometabolic risk factors, such as childhood obesity, hypertension, abnormal cholesterol levels, and elevated insulin. Notably, these risks may persist from childhood through adolescence and into adulthood [70]. Therefore, reducing sedentary behavior and increasing physical activity during childhood is essential for promoting healthy growth.

Finally, this study also found that age, sleep duration, and parental support for physical activity play significant roles in predicting children’s physical literacy. The significance of age in the models suggests that physical literacy levels can change significantly as children grow older, highlighting the need to consider age when planning early interventions. Moreover, sleep duration was identified as an important predictor, with children in the“high physical literacy” group getting slightly more sleep than those in the “needs attention” group (*P* = 0.008). This finding suggests that adequate sleep contributes to better physical literacy, consistent with Lemes et al., who found that well-rested children are more willing to engage in physical activities [52], thereby enhancing their physical literacy. However, the extent and mechanisms of how sleep affects physical literacy still require further exploration. The connection between sleep and physical literacy likely involves several factors. On one hand, sleep is crucial for physiological and cognitive functions, with studies confirming that a lack of sleep impairs motor performance by disrupting the autonomic nervous system and reducing coordination [71]. On the other hand, psychological factors such as stress, anxiety, and depression are closely linked to sleep problems [72], potentially affecting children’s mental state and motivation during physical activities, which could lower their overall physical literacy. Additionally, parental support was a key factor in predicting physical literacy. Hinkley et al. pointed out that sociocultural factors play a significant role in shaping preschool children’s physical activity levels and patterns [73]. Further research showed that highly supportive parenting helps children build positive beliefs and values related to physical activity [74]. A meta-analysis by Yao and Rhodes also confirmed that parental support and role modeling are strongly linked to children’s physical activity [75]. These findings align with our study’s results, further emphasizing the crucial role of parental support in determining children’s physical literacy and highlighting the family as a key environment for fostering healthy behaviors. It is important to note that although our analysis found that the proportion of children in urban–rural fringe areas classified into the ‘Needs Attention’ group was slightly higher than that in main urban districts, the difference was not statistically significant. This may be due to the strong support of the national Rural Revitalization Strategy in recent years [76], under which local governments have continuously improved facilities and programs for children’s physical activities in urban–rural fringe areas, thereby narrowing the gap between main urban districts and urban–rural fringe areas in the development of children’s physical literacy.

### Limitations and future directions

This study has several limitations that should be acknowledged. First, the data were collected from specific regions within Fujian Province, which may limit generalizability. Future studies should expand the geographic scope to validate the results. Second, physical literacy was measured using self-reported data, which is susceptible to recall bias or social desirability effects. Although self-reporting is cost-effective and convenient for large-scale surveys, future research should combine subjective and objective measures to improve reliability. Finally, while 16 influencing factors were included in this study, they may not capture all potential predictors. Future research should broaden the range of predictors to support the development of more comprehensive predictive models of children’s physical literacy.

## Conclusions

Our study revealed that the overall physical literacy levels of children aged 4–6 in Fujian Province are relatively low. Among the four domains, the social domain scored the highest, showing that these children have relatively mature social behaviors and cooperative skills in physical activities. However, the physical domain lagged behind, indicating that there’s substantial room for improvement in their physical fitness and motor skills. By applying multiple machine learning models, including LR, SVM, XGBoost, RF, and Ensemble Learning, this study systematically analyzed the key factors influencing children’s physical literacy. The findings revealed that mother’s exercise frequency, family exercise environment, and children’s MVPA time were the most important predictive factors, significantly impacting physical literacy levels. Additionally, the Ensemble Learning model outperformed the others, particularly in AUC, specificity, accuracy, and F1 score, demonstrating its superiority over single models. This validates the effectiveness of Ensemble Learning in handling complex datasets.

This study makes several innovative contributions. First, it fills a gap in research on physical literacy among younger children aged 4–6 by applying multiple machine learning models to analyze the impact of family environment and maternal exercise frequency—an area that hasn’t been fully explored in existing literature. Second, this study is the first to apply an Ensemble Learning method to predict physical literacy in children aged 4–6. By combining SVM, XGBoost, and RF models, it significantly enhances prediction accuracy and robustness, providing a more precise tool for understanding the complex relationships in children’s physical literacy. The study also took a multidimensional analysis approach, systematically examining individual factors like gender, age, and body type, while also considering family factors like parental education, exercise frequency, and support attitudes. This comprehensive framework offers new research pathways and theoretical support for understanding and improving physical literacy in preschool children aged 4–6.
